# Biological mode of action of a nitrophenolates-based biostimulant: case study

**DOI:** 10.3389/fpls.2014.00713

**Published:** 2014-12-16

**Authors:** Arkadiusz Przybysz, Helena Gawrońska, Janina Gajc-Wolska

**Affiliations:** ^1^Laboratory of Basic Research in Horticulture, Faculty of Horticulture, Biotechnology and Landscape Architecture, Warsaw University of Life Sciences – SGGWWarsaw, Poland; ^2^Department of Vegetable and Medicinal Plants, Faculty of Horticulture, Biotechnology and Landscape Architecture, Warsaw University of Life Sciences – SGGWWarsaw, Poland

**Keywords:** biomass accumulation, efficiency of photosynthetic apparatus, growth and development, nitrophenolates, water status, yield, yield parameters

## Abstract

The challenges facing modern plant production involve (i) responding to the demand for food and resources of plant origin from the world's rapidly growing population, (ii) coping with the negative impact of stressful conditions mainly due to anthropopressure, and (iii) meeting consumers' new requirements and preferences for food that is high in nutritive value, natural, and free from harmful chemical additives. Despite employing the most modern plant cultivation technologies and the progress that has been made in breeding programs, the genetically-determined crop potential is still far from being fully exploited. Consequently yield and quality are often reduced, making production less, both profitable and attractive. There is an increasing desire to reduce the chemical input in agriculture and there has been a change toward integrated plant management and sustainable, environmentally-friendly systems. Biostimulants are a category of relatively new products of diverse formulations that positively affect a plant's vital processes and whose impact is usually more evident under stressful conditions. In this paper, information is provided on the mode of action of a nitrophenolates-based biostimulant, Atonik, in model species and economically important crops grown under both field and controlled conditions in a growth chamber. The effects of Atonik on plant morphology, physiology, biochemistry (crops and model plant) and yield and yield parameters (crops) is demonstrated. Effects of other biostimulants on studied in this work processes/parameters are also presented in discussion.

## Introduction

The challenge facing modern plant production nowadays is to respond to the increasing demand for food and resources of plant origin by the world's rapidly growing population. Yield is negatively affected by various adverse environmental conditions and increasing anthropopression and despite employing the most modern plant cultivation technologies and the progress being made in breeding programs, the genetically-determined crop potential is still far from being fully exploited. According to Bray et al. (2000), stresses can reduce average productivity by 65–87%, depending on the crop. This consequently makes plant production less profitable for farmers and less attractive for consumers.

Biostimulants *syn*. biostimulators are a category of relatively new products of diverse formulations that positively affect a plant's vital processes (Calvo et al., 2014), usually more evident under stressful conditions, by increasing a plant's tolerance to stresses and repairing damage caused by unfavorable conditions.

Biostimulants may be of natural or synthetic origin and consist of various organic and inorganic components. Among naturally derived biostimulants are preparations based on free amino acids, extracts from seaweed and fruit, effective microorganisms, humic substances, and chitosan (Calvo et al., 2014). Synthetic biostimulants are composed, among others, of plant growth regulators, phenolic compounds, inorganic salts, essential elements, and other substances that have stimulating properties for plants.

Although the term “biostimulant” has been used for many years, it is still not fully defined. The European Biostimulant Industry Council (EBIC) describes biostimulants as a preparations “… containing substance(s) and/or micro-organisms whose function, when applied to plants or the rhizosphere is to stimulate natural processes to enhance/benefit nutrient uptake, nutrient efficiency, tolerance to abiotic stress, and crop quality….” Biostimulants do not replace, but rather complement plant protection products and fertilizers. They have no direct action against pests and they operate through different mechanisms than fertilizers, regardless of the occasional presence of nutrients in these products (http://www.biostimulants.eu).

It is impossible to suggest one common mode of action for all biostimulants, therefore this work focused on Atonik, known as Chapperone (USA) or Asahi SL (Poland). Atonik is a Japanese synthetic biostimulant composed of three phenolic compounds: sodium para-nitrophenolate PNP (0.3%), sodium ortho-nitrophenolate ONP (0.2%) and sodium 5-nitroguaiacolate 5NG (0.1%), and water. Atonik has been used successfully for many years in the cultivation of most important crops worldwide. Its positive effect on yield is already well proven (Djanaguiraman et al., 2004a, 2009; Bynum et al., 2007; Grajkowski and Ochmian, 2007; Budzyński et al., 2008; Černý et al., 2008; Kositorna and Smoliński, 2008; Kozak et al., 2008a; Malarz et al., 2008; Michalski et al., 2008; Sawicka and Mikos-Bielak, 2008), but knowledge about its mode of action has, until this study, been fragmented, not covered thoroughly in literature, and sometimes even controversial. Early works described some of the potential positive properties of Atonik. It has been shown that the nitrophenolates making up this biostimulant increase cytoplasm streaming (Yamaki et al., 1953; Wilson and Kaczmarek, 1993). Plants treated with nitrophenolates have greater inhibition of IAA oxidase, which ensures a higher activity of naturally synthesized auxins (Stutte and Clark, 1990). The phosphorylated form of para-nitrophenolate enhances IAA activity when used as a substrate for phosphatases *via* increased high-affinity binding sites of IAA (Davies, 1987) and could be as effective as ATP (Koizumi et al., 1990). According to Stutte et al. (1987), plants exposed to nitrophenolates uptake more nutrients from the medium. Furthermore, Sharma et al. (1984) showed a significant increase in the activity of nitrate reductase, an important enzyme in nitrogen metabolism.

More recent studies prove that Atonik positively affects various processes controlling plant growth, development and productivity. Biostimulant-treated plants are more advanced in growth and development (Djanaguiraman et al., 2005b; Gulluoglu et al., 2006; Kozak et al., 2008a; Borowski and Blamowski, 2009) and accumulate more biomass (Gruszczyk and Berbeć, 2004; Djanaguiraman et al., 2005a, 2009; Kołodziej, 2008). Atonik increases the intensity of photosynthesis (Borowski and Blamowski, 2009) and transpiration rate, but usually without a reduction in relative water content (Wróbel and Woźniak, 2008; Borowski and Blamowski, 2009). The positive effects of Atonik are much more evident when plants are grown under adverse conditions. It has been found that biostimulants play a protective role against various abiotic stresses, such as low or high temperatures, drought, heavy metals, and salinity (Gulluoglu et al., 2006; Gawrońska et al., 2008; Wrochna et al., 2008; Borowski and Blamowski, 2009). Moreover, some results have indicated that if plants were grown under optimal conditions, the positive effect of this preparation might not be recorded (Budzyński et al., 2008; Księżak, 2008).

However, the works presented above individually only cover a narrow range of processes and/or parameters. This paper provides the first comprehensive study of the Atonik mode of action and demonstrates the effects of biostimulant on yield and its components, plant morphology, physiology and biochemistry in the model plant *Arabidopsis thaliana* L. and some crops that are economically important (*Brassica napus* L. *var. oleifera* and *Cucumis sativus* L.).

## Materials and methods

The experiments were carried out on crops: oilseed rape and cucumber and *A. thaliana* used as model plant. Plants were grown in field conditions and growth chambers under optimal, drought or noble metal stresses. Concentrations of Atonik and the number of its applications were first determined in preliminary studies in order to ensure a stimulative/protective effect of the biostimulant in particular species and growing conditions.

### Effect of Atonik on field-grown oilseed rape plants

#### Plant material and growing conditions

Oilseed rape *cv*. “Lisek” plants were cultivated in the 2007 and 2008 growing seasons in the experimental field in Chylice of the Warsaw University of Life Sciences—SGGW. The field is situated 105 m above sea level and located at 22°33'25” N and 52°05'71” E. The 30-year average annual temperature and rainfall are 7.8°C (12.8°C during the growing seasons) and 592 mm (448 mm during the growing seasons) respectively. The soil (black degraded, composed of loamy sand) is classified as average good, with a 0.8–1.6% content of organic matter and pH 6.0–6.2. Experiments were conducted in completely randomized blocks in four replicates (plots of 18 or 14.4 m^2^ in 2007 and 2008, respectively). The seeds were sown at a spacing of 30 × 6.5 cm. For the measurements, five plants from each plot were chosen. In the 2007 growing season the experimental plants were grown in 25 L pots filled with soil taken from the particular plots, and the pots were placed (buried) on the appropriate plots, following a statistical design. Routine agricultural practices recommended for this species and location were employed. Both vegetative seasons were characterized by similar growing conditions, the only exception was a strong late spring frost in 2007. Atonik was applied in spring as a single (BBCH 29–31) or double (BBCH 29–31 and 51) foliar spray in a concentration of 0.2% v/v in 300 L ha^−1^. NPK fertilizers were applied as 194 kg N ha^−1^ (34—autumn, 160—spring), 80 kg P ha^−1^ and 120 kg K ha^−1^.

#### Measured parameters/processes

One (2007) or three (2008) weeks after the Atonik application, the following parameters were measured: (i) plant gas exchange: intensity of photosynthesis and transpiration, stomatal resistance (Photosynthesis System LICOR 6200, Lincoln, NE, USA), (ii) chlorophyll content (CCM-200, OPTI-SCIENCES, USA) and (iii) chlorophyll *a* fluorescence (Handy PEA, Hansatech, UK). The measurements were performed for 9 (2007) and 10 (2008) weeks. After harvest, (i) the height of the plants was measured, (ii) the number of leaves, primary laterals, pods and seeds in pods were counted, and (iii) the accumulation of biomass (after drying at 105°C for 2 h and then at 75°C for 48 h) and yield of seeds (*via* weighing of air dry seeds) were recorded.

### Effect of Atonik on field-grown cucumber plants

#### Plant material and growing conditions

Cucumber *cvs*. “Octopus F1” (Syngenta Seeds), Opera F1 and Sonate F1 (both Rijk Zwaan) plants were cultivated in the 2012 growing season in the experimental field of the Department of Vegetable and Medicinal Plants at Wilanów, Poland. Plants were grown in deep medium-heavy alluvial soil (classified as good) with a 1.9–2.3% content of organic matter and pH 6.0–6.5. The experiment was arranged in a two-factor split-plot design with four replicates (plots of 6 m^2^). Seeds were sown manually on 14 May into plastic pots of 8 cm diameter filled with peat substrate. On 24 May, when the plants had 1–2 leaves, seedlings were planted in the field at a spacing of 30 × 150 cm. There were 14 plants in the plot. Atonik was applied as a foliar spray (12 and 27 June and 27 July) in a concentration of 0.1% v/v in 500 L ha^−1^. Control plants were treated with water. During the period of water shortage, plants were T-Tape irrigated. The soil content of N, P, and K was kept at the optimum level, with fertilizers applied to equal the average of 150 kg N ha^−1^ (60 kg N side dressing), 50 kg P ha^−1^ and 190 kg K ha^−1^. The harvest was carried out successively, twice a week (13 times), starting from the middle of September.

#### Measured parameters/processes

At harvest, the total and marketable yield was recorded. Yield quality was evaluated by determining the content of: (i) dry matter (drying to constant weight at 105°C), (ii) sugars (Luff–Schoorl method), (iii) vitamin C (titration with Tillmans' method), (iv) nitrates (spectrophotometer Tecator Fiastar 5010 at wavelength 540 nm), (v) phosphorus (spectrophotometer Shimadzu 1700 at wavelength 460 nm), (vi) potassium, and (vii) calcium (both using flame spectrophotometer Sherwood Model 410). Marketable fruits were graded according to the Polish standard PN-85/R-75359 into two pickling grades of (i) 6–10 cm long with a diameter of 2.5–4.5 cm and (ii) 9–15 cm long with a diameter of 4.5–5.5 cm.

### Effect of Atonik on *A. thaliana* plants grown under optimal, drought, and Pt stress

#### Plant material and growing conditions

*A. thaliana* Col 4 seeds (Lehle Seeds, Round Rock, TX, USA) were sown onto multiplates filled with substrate (Universal Kronenerde soil and sand in the proportion 2:1 v/v). Uniform, 6-week-old seedlings were transplanted to (i) pots (Ø 10 cm) containing the same substrate or (ii) hydroponics culture filled with 0.3 dm^3^ of a Hoagland solution (Arnon and Hoagland, 1940) modified by Siedlecka and Krupa (2002). The nutrient solution was continuously aerated and renewed weekly. Plants were grown in growth chambers (Simez Control s.r.o. Vsetin, Czech Republic) at 22/18°C with a photoperiod 8/16 h day/night, irradiance of 250–280 μmol m^−2^s^−1^ PAR and relative humidity of 60%.

#### Drought stress

Before drought treatment, the maximum water capacity (MWC) of the substrate was determined. Drought stress was imposed on the soil as a result of a daily limited water supply *via* pot weighing to the levels of 50, 40, 30, and 20% of MWC (three experiments) or 45 and 25% of MWC (two experiments). Depending on the experiment, the combination consisted of 6–12 plants. On the day on which the substrate attained the desired MWC, the plants were treated once with Atonik as a foliar spray at a concentration of 0.1% (with an amount of water equal to 300 L ha^−1^ in the field conditions) and grown for a further 4 weeks. Control plants were cultivated at 60 or 65% MWC (optimal water conditions) and sprayed with distilled water.

#### Pt stress

During the first week the nutrient solution was used at half strength and thereafter the complete composition of macro- and microelements was supplied. Two weeks after plants were transplanted to hydroponics, during the nutrient solution change, Pt and Atonik were added. Pt, in oxidation state II, was added at concentrations of 2.5, 25, and 50 μM in the form of [Pt(NH_3_)_4_](NO_3_)_2_. Atonik was added at a concentration of 0.005% v/v. After treatment, the plants were grown for a further 3 weeks. In total, three experiments were carried out, with 5–6 plants per combination. Control plants were grown in Pt and Atonik-free medium.

#### Measured parameters/processes

During plant growth the following parameters were measured: (i) plant gas exchange: intensity of photosynthesis and transpiration, stomatal resistance (Photosynthesis System LICOR 6200, Lincoln, NE, USA), (ii) chlorophyll content (CCM-200, OPTI-SCIENCES, USA), (iii) chlorophyll *a* fluorescence (Handy PEA, Hansatech, UK), and (iv) water uptake (*via* daily pot weighing). At harvest, sub-samples were collected for (i) relative water content (RWC, *via* weighing) and (ii) membrane injury (conductometrically, MultiLevel 1, WTW, Germany) and data recorded on (iii) the height of plants, (iv) length and number of inflorescences, (v) number of pods, (vi) number and area of leaves (Leaf Area, Root Length and Image Analyzing System, Skye, UK), and (vii) biomass accumulated by the whole plants and particular organs (after drying at 105°C for 2 h and then at 75°C for 48 h).

### Statistics

The number of replications, depending on the parameter, was between 3 and 36, and is indicated in the specific tables or figures. Differences between the combinations were evaluated with one or two-factor analysis of variance by LSD (Student's *t*-test) or HSD (Tukey test) at α = 0.05. The presented data are mean ± SE (where indicated).

## Results

### Effect of Atonik on field-grown oilseed rape plants

Atonik-treated plants in the 2007 season were taller than the control, and produced slightly more pods (0.1–4.1%) and seeds in pods (0.9–2.8%) (Table 1). On the other hand these plants developed a lower number of primary laterals. In the 2008 season, the biostimulant had no effect on the plants' height. Regardless of whether Atonik was used once or twice, the number of laterals (9.5–12.5%) and seeds (0.7–2%) was greater. Only the single spray increased the number of pods (8.1%) (Table 1).

**Table 1:** **Effect of Atonik on selected morphological parameters of oilseed rape plants**.

**Combination**	**Height (cm plant^−1^)**	**Number of (plant^−1^)**		
		**Laterals**	**Pods**	**Seeds**
**2007 GROWING SEASON**				
Control	108.90 (±1.38)	5.55 (±0.27)	100.45 (±5.50)	15.92 (±0.14)
Atonik 1×	118.89^*^ (±1.81)	5.42 (±0.21)	104.58 (±5.17)	16.06 (±0.13)
Atonik 2×	111.28 (±1.43)	5.22 (±0.24)	100.56 (±7.27)	16.37 (±0.15)
**2008 GROWING SEASON**				
Control	162.10 (±1.29)	10.05 (±0.25)	253.00 (±8.50)	25.46 (±0.18)
Atonik 1×	161.63 (±1.16)	11.31 (±0.26)	273.63 (±11.87)	25.98 (±0.42)
Atonik 2×	160.05 (±2.13)	11.00 (±0.24)	250.08 (±8.91)	25.65 (±0.71)

*Presented data are Mean ± SE, n = 20*.

* *Values differ significantly at α = 0.05 as determined by LSD of t-Student test*.

The fresh weight of Atonik-treated plants in 2007 was 12.5% higher than that of the control and in the case of dry matter Atonik contributed to an increase of between 11.9–23.7% (Table 2). The fresh weight and dry matter of stem and pods with seeds were also greater. Higher values were obtained for a single spray. In the next season the positive influence of Atonik on accumulated biomass was less evident and was recorded after a single spray only. Atonik slightly increased the fresh weight and dry matter of the aboveground part, the main stem and pods with seeds. The weight of the laterals was adversely affected. The yield of plants sprayed once with Atonik exceeded the control by 35% (2007) or by just 3.6% (2008). After the double application no positive effect or even reduction was noted (Table 2).

**Table 2:** **Effect of Atonik on biomass accumulation and seed yield in oilseed rape plants**.

**Measured parameter**	**Combination**	**g plant ^−1^**				
		**Whole plant**	**Pods with seeds**	**Main stem**	**Laterals**	**Yield**
**2007 GROWING SEASON**						
Fresh weight	Control	37.42 (± 1.96)	14.00 (± 0.85)	19.69 (± 1.02)	3.74 (± 0.37)	
	Atonik 1×	42.10 (± 2.79)	16.15 (± 1.17)	22.36 (± 1.36)	3.59 (± 0.34)	
	Atonik 2×	42.18 (± 2.78)	15.94 (± 1.97)	21.21 (± 1.43)	5.02 (± 0.50)	
Dry matter	Control	9.60 (± 0.51)	4.33 (± 0.27)	4.21 (± 0.20)	1.03 (± 0.10)	2.29 (± 0.16)
	Atonik 1×	11.87 (± 0.66)	5.52 (± 0.36)	5.33 (± 0.26)	1.02 (± 0.08)	3.09 (± 0.23)
	Atonik 2×	10.75 (± 0.67)	4.60 (± 0.39)	4.93 (± 0.26)	1.21 (± 0.10)	2.32 (± 0.24)
**2008 GROWING SEASON**						
Fresh weight	Control	147.86 (± 6.24)	59.86 (± 2.60)	62.84 (± 2.55)	25.15 (± 1.71)	
	Atonik 1×	154.26 (± 5.68)	61.28 (± 2.59)	69.69 (± 2.32)	23.28 (± 1.31)	
	Atonik 2×	136.82 (± 5.44)	50.89 (± 2.01)	63.58 (± 2.32)	22.36 (± 1.58)	
Dry matter	Control	68.46 (± 2.50)	42.12 (± 1.55)	15.75 (± 0.53)	10.59 (± 0.58)	24.69 (± 0.94)
	Atonik 1×	70.18 (± 2.19)	44.11 (± 1.44)	16.34 (± 0.37)	9.73 (± 0.46)	25.58 (± 1.03)
	Atonik 2×	62.55 (± 2.42)	38.03 (± 1.55)	15.33 (± 0.45)	9.18 (± 0.48)	21.93 (± 0.93)

*Presented data are Mean ± SE, n = 20*.

In the 2007 season, irrespective of the number of treatments, Atonik increased photosynthesis intensity (1–22%) and this effect lasted up to 7 weeks following the first spray (Table 3). In the following year the positive effect on this process remained for 4 weeks (3.6–20.3%). In the 2007 season, the sprayed plants were usually characterized by a higher intensity of transpiration and lower stomatal resistance. In contrast to this, in the 2008 season the effect of Atonik on these parameters was ambiguous. The total chlorophyll content in both growing seasons was, with a few exceptions, higher in biostimulant-treated plants (Table 3).

**Table 3:**
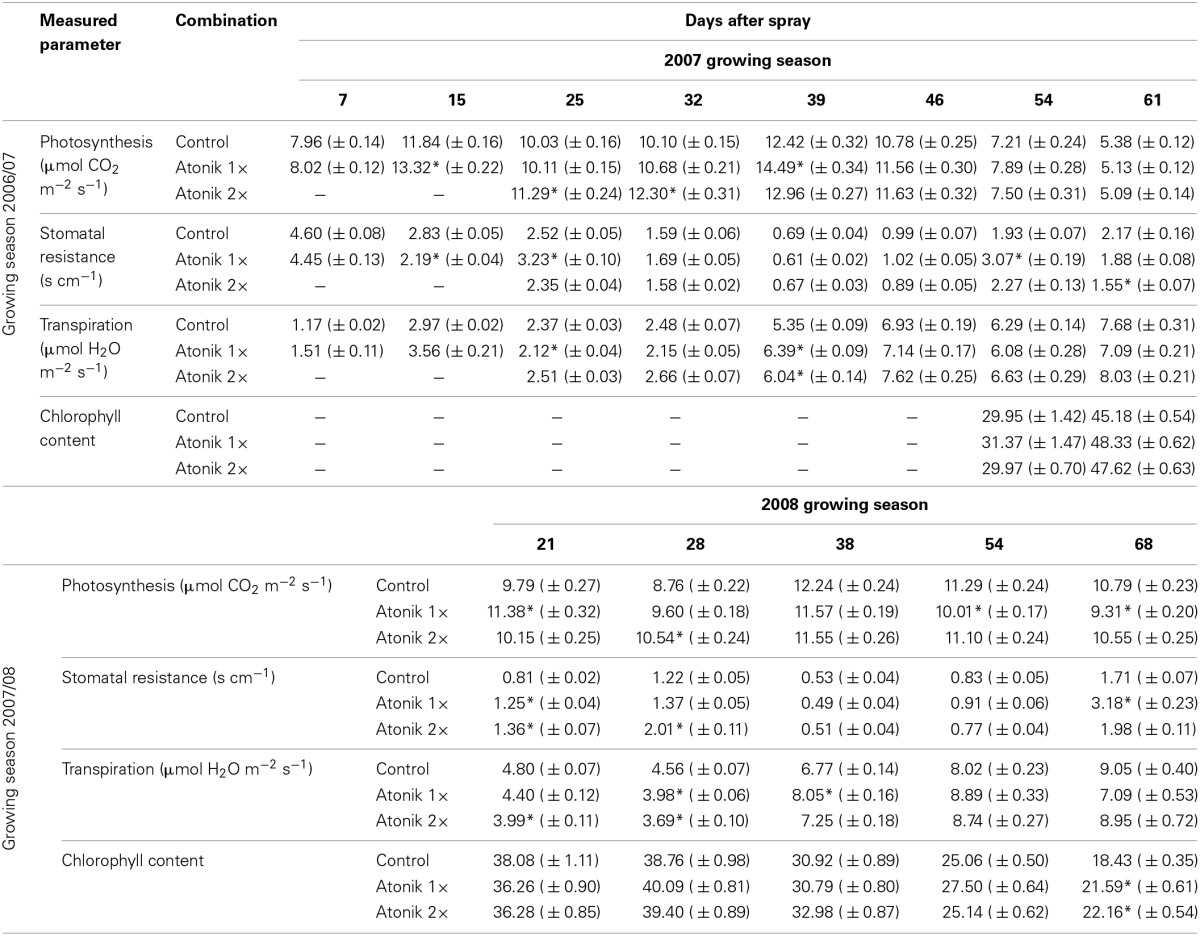
**Effect of Atonik on the intensity of photosynthesis and transpiration, stomatal conductance, transpiration and chlorophyll content in oilseed rape plants**.

*Presented data are Mean ± SE, n = 24 or 36 (chlorophyll content)*.

^*^Values differ significantly at α = 0.05 as determined by LSD of t-Student test.

**Figure 1:**
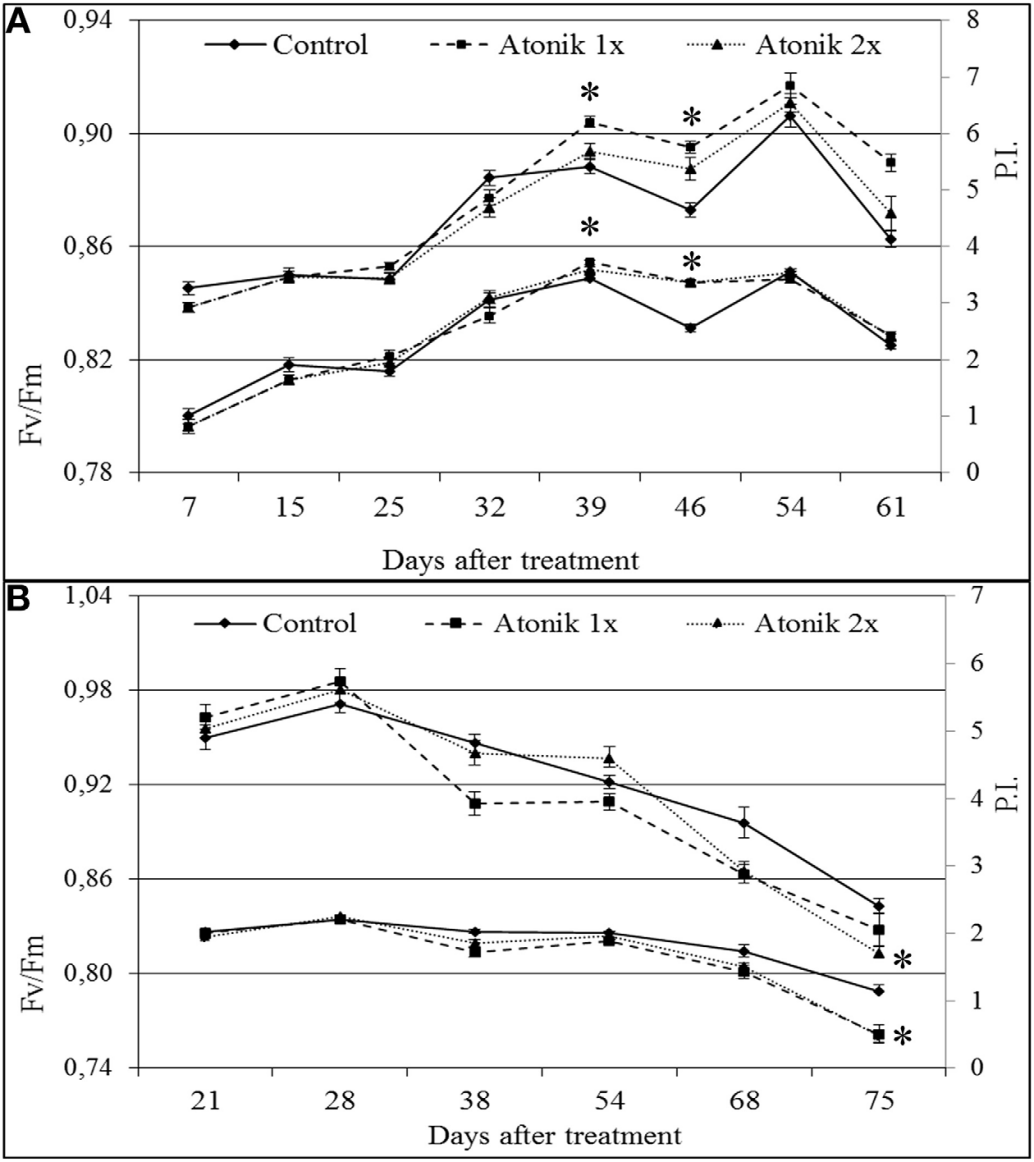
**Effect of Atonik on selected parameters of chlorophyll *a* fluorescence (Fv/Fm and PI) in oilseed rape *cv***. Lisek plants grown under field conditions during the 2007 **(A)** and 2008 **(B)** vegetation seasons. Presented data are mean ± SE, *n* = 24. ^*^Values differ significantly at α = 0.05 as determined by LSD of *t*-Student test.

Measurements of chlorophyll *a* fluorescence showed that in the 2007 season Atonik did not affect Fv/Fm (maximum quantum efficiency of Photosystem II) and P.I. (Performance Index) up to the late spring frost (−4.2°C) that occurred between the 36 and 39th day after the first application of the biostimulant (Figure 1A). Following the frost, a lowering in the Fv/Fm and P.I. values in the control was recorded, while in the treated plants they did not change. Moreover, the positive effect on P.I. remained for the next 22 days. The values of these parameters in the 2008 season during the 8 weeks after the first spray were similar between the treated and untreated plants. Starting from week 10, a reduction in these parameters after the application of Atonik was noted (Figure 1B).

### Effect of Atonik on field-grown cucumber plants

There was no significant effect of the Atonik on total or marketable yield, or any interactions of both traits examined (Table 4). Slightly increased yields after biostimulant treatment were recorded only for the cultivar Octopus F_1_. Yields of fruits were significantly related to the cultivar. The highest values of fruit mass were recorded for cultivar Sonate F_1_ and the lowest for Octopus F_1_. On average, for all the examined cultivars, the content of dry matter and soluble solids were significantly higher after treatment with the biostimulant. When the cultivars were examined separately, it came out that dry content increased by Atonik, except in Opera F_1_. Soluble solids were always higher in plants sprayed with the biostimulant. The content of nitrates was higher on average in plants treated with Atonik. The exception was the Sonate F_1_ cultivar. In plants sprayed with the biostimulant, a higher content of phosphorus was recorded. The content of potassium was only significantly affected by the cultivar and the highest was found in Octopus F_1_, while the lowest was in Sonate F_1_. The content of calcium was affected by both the biostimulant and the cultivar. The effect of Atonik on this parameter was adverse and the Sonate F_1_ cultivar was characterized as having the greatest content of calcium and Opera F_1_ the lowest (Table 4).

**Table 4:** **Effect of Atonik on the total and marketable yield of cucumber fruit, content of dry matter, soluble solids, nitrates, phosphorus, potassium, and calcium**.

**Cultivar**	**Total yield (kg m^−2^)**		**Mean for cultivar**	**Marketable yield (kg m^−2^)**		**Mean for cultivar**
	**Control**	**Atonik**		**Control**	**Atonik**	
Octopus F_1_	6.43 b^a^	7.52 a	6.97 b	3.94 b	4.76 b	4.35 b
Opera F_1_	7.74 a	7.17 a	7.45 ab	5.71 a	5.20 a	5.45 a
Sonate F_1_	8.35 a	8.26 a	8.30 a	6.05 a	5.95 a	6.00 a
Mean for treatments	7.51 a	7.65 a		5.23 a	5.30 a	
	**Dry matter (%)**			**Soluble solids (%)**		
Octopus F_1_	4.22 a	4.75 a	4.48 a	4.10 a	4.20 a	4.15 a
Opera F_1_	4.94 a	4.74 a	4.84 a	4.10 a	4.47 a	4.28 a
Sonate F_1_	4.50 a	5.20 a	4.85 a	4.13 a	4.23 a	4.18 a
Mean for treatments	4.56 a	4.90 a		4.11 b	4.30 a	
	**Nitrates (mg 100 g^−1^ FW)**			**Phosphorus (mg 100 g^−1^ FW)**		
Octopus F_1_	12.96 c	13.99 b	13.47 a	16.14 b	18.24 a	17.19 a
Opera F_1_	13.10 b	15.01 a	14.05 a	15.93 b	17.82 b	16.87 ab
Sonate F_1_	13.79 b	13.23 b	13.51 a	11.81 c	12.29 c	12.05 c
Mean for treatments	13.28 b	14.08 a		14.63 b	16.11 a	
	**Potassium (mg 100 g^−1^ FW)**			**Calcium (mg 100 g^−1^ FW)**		
Octopus F_1_	219.84 a	215.78 a	217.81 a	6.96 b	5.77 b	6.36 b
Opera F_1_	209.79 a	209.38 a	209.58 a	6.03 b	6.16 b	6.09 b
Sonate F_1_	196.35 b	198.88 b	197.61 b	13.67 a	13.67 a	13.67 a
Mean for treatments	208.66 a	208.0 a		8.89 a	8.53 a	

*Presented data are mean, n = 3*.

a *Data in columns followed by the same letter do not differ significantly as based on the HSD of the Tukey test at confidence level of 95%*.

### Effect of Atonik on *A. thaliana* plants grown under optimal, drought, and Pt stresses

#### Optimal and drought conditions

**Figure 2:**
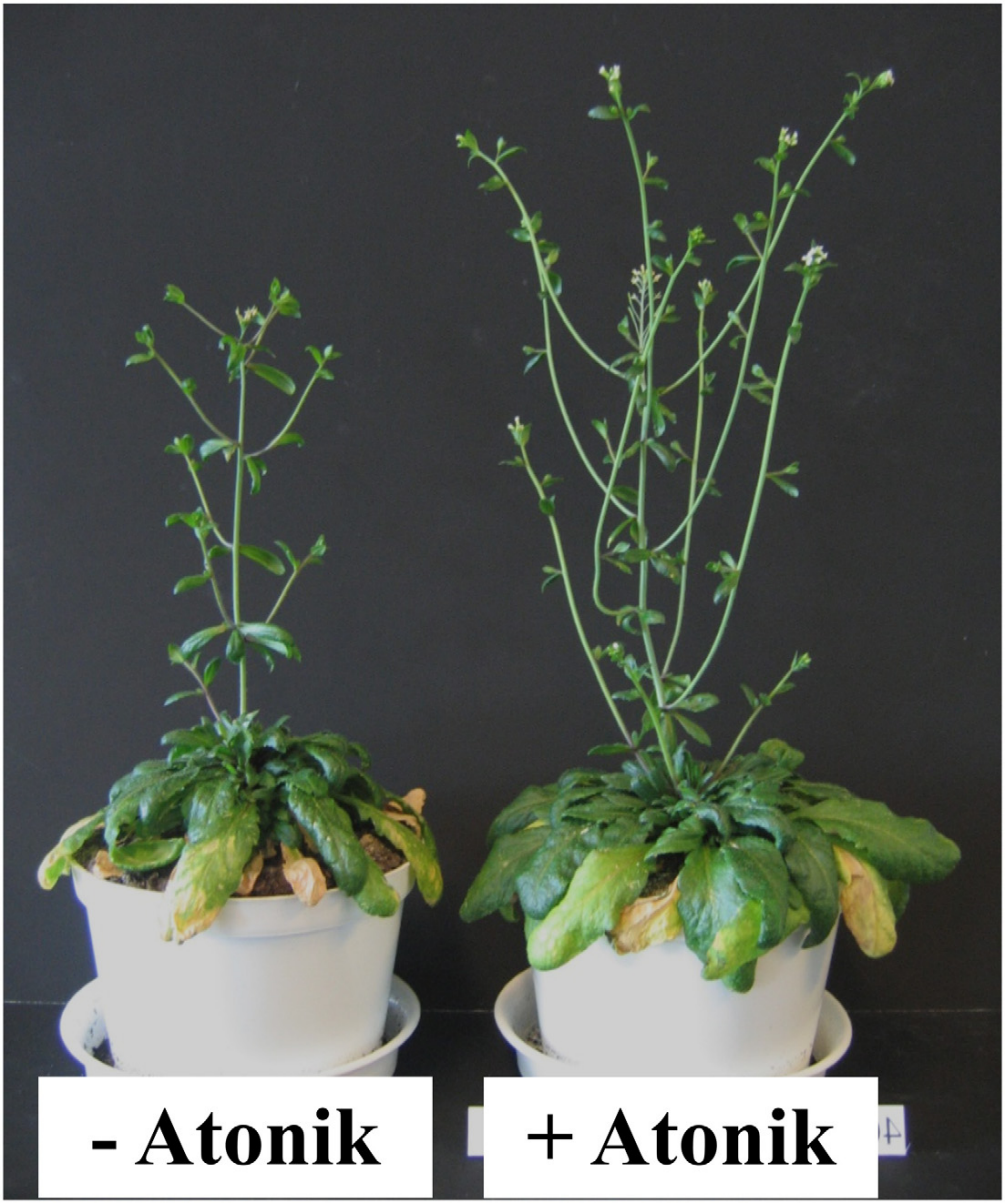
**Effect of Atonik on growth and development of *A. thaliana* plants grown under drought stress conditions (40% MWC)**.

Atonik had a positive effect on *A. thaliana* grown in optimal conditions and clearly diminished the negative impact of drought (Figure 2). Plants sprayed with Atonik were taller and developed more inflorescences (by 14–56%) and pods (by 93–450%) (Table 5). In 20 and 30% of MWC their number reduced. Leaf area was always greater in Atonik-treated plants, and this increase ranged between 3–43% (Table 5).

**Table 5:** **Effect of Atonik on selected morphological parameters of *A. thaliana* plants grown under optimal and drought stress conditions**.

**MWC (%)**	**Height (cm plant^−1^)**		**Number of (inflorescence plant^−1^)**		**Number of (pod plant^−1^)**		**Leaf area (cm^2^ plant^−1^)**	
	**−Atonik**	**+Atonik**	**−Atonik**	**+Atonik**	**−Atonik**	**+Atonik**	**−Atonik**	**+Atonik**
60	34.95 (± 2.42)	42.92 (± 1.17)	27.50 (± 3.18)	43.00 (± 5.86)	12.00 (± 2.09)	41.50 (± 9.83)	164.16 (± 9.53)	191.51 (± 4.60)
50	24.67 (± 1.91)	29.17 (± 1.60)	20.50 (± 2.93)	23.50 (± 1.53)	7.75 (± 2.00)	15.00 (± 6.84)	130.22 (± 10.32)	134.25 (± 4.81)
40	20.95 (± 0.61)	26.12 (± 2.07)	18.50 (± 2.73)	26.25 (± 4.13)	0.50 (± 0.25)	2.75 (± 0.69)	104.06 (± 2.15)	110.03 (± 5.23)
30	14.12 (± 0.71)	21.47 (± 1.49)	18.50 (± 1.49)	25.75 (± 2.68)	1.75 (± 0.88)	1.00 (± 0.29)	77.02 (± 4.20)	110.33^*^ (± 3.26)
20	11.72 (± 0.82)	13.37 (± 1.29)	15.25 (± 1.66)	15.00 (± 1.24)	0.50 (± 0.25)	1.50 (± 0.75)	81.02 (± 4.07)	88.57 (± 4.46)

*Data are Mean ± SE, n = 5*.

* *Values differ significantly at α = 0.05 as determined by LSD of t-Student test*.

*A. thaliana* treated with Atonik produced more biomass and this was true for optimal conditions and every level of drought stress (Table 6). The increase of biomass accumulation recorded ranged between 2.5–46 and 1–47%, respectively for fresh weight and dry matter. The positive effect of Atonik was more evident in the case of generative organs (Table 6).

**Table 6:** **Effect of Atonik on the fresh matter of the whole aboveground part, inflorescence and rosette of *A. thaliana* plants grown under optimal and drought stress conditions**.

**MWC (%)**	**Aboveground part**		**Inflorescence with pods**		**Rosette**	
	**−Atonik**	**+Atonik**	**−Atonik**	**+Atonik**	**−Atonik**	**+Atonik**
**FRESH WEIGHT g PLANT^−1^**						
60	18.28 (± 0.97)	23.97 (± 0.62)	5.49 (± 0.57)	8.28 (± 0.79)	12.78 (± 0.49)	15.69 (± 0.48)
50	16.07 (± 0.93)	16.48 (± 0.89)	4.96 (± 0.29)	4.74 (± 0.33)	11.12 (± 0.94)	11.74 (± 0.89)
40	13.14 (± 0.56)	16.06 (± 0.31)	3.79 (± 0.38)	4.36 (± 0.33)	9.35 (± 0.20)	11.70 (± 0.38)
30	9.70 (± 0.20)	14.15^*^ (± 0.20)	2.36 (± 0.15)	4.11 (± 0.41)	7.34 (± 0.08)	10.04^*^ (± 0.21)
20	9.32 (± 0.40)	10.36 (± 0.47)	1.34 (± 0.16)	2.12 (± 0.23)	7.97 (± 0.24)	8.50 (± 0.24)
**DRY MATTER g PLANT^−1^**						
60	2.03 (± 0.10)	2.58 (± 0.05)	0.68 (± 0.07)	1.07 (± 0.09)	1.35 (± 0.05)	1.51 (± 0.05)
50	1.94 (± 0.11)	1.96 (± 0.10)	0.61 (± 0.05)	0.61 (± 0.04)	1.33 (± 0.09)	1.35 (± 0.09)
40	1.68 (± 0.08)	1.99 (± 0.06)	0.48 (± 0.05)	0.59 (± 0.05)	1.20 (± 0.03)	1.41 (± 0.05)
30	1.19 (± 0.06)	1.76 (± 0.01)	0.30 (± 0.02)	0.56 (± 0.04)	0.97 (± 0.00)	1.21 (± 0.05)
20	1.25 (± 0.02)	1.35 (± 0.05)	0.17 (± 0.02)	0.30 (± 0.03)	1.07 (± 0.00)	1.05 (± 0.04)

*Data are Mean ± SE, n = 5*.

* *Values differ significantly at α = 0.05 as determined by LSD of t-Student test*.

The efficiency of the photosynthetic apparatus of *A. thaliana* plants was positively affected by the biostimulant (Table 7). The intensity of photosynthesis was usually higher in Atonik-treated plants and this increase ranged from 0.5 to as high as 55.5%. The greater intensity of photosynthesis corresponded well with the significantly lowered stomatal resistance. The effect of Atonik on chlorophyll content in *A. thaliana* was not uniform. Measurements taken seven days after the treatment revealed the biostimulant's positive effect on this parameter, but 14 days after the Atonik application a greater chlorophyll content was recorded in 50 and 40% of MWC. Atonik also influenced parameters of chlorophyll *a* fluorescence, especially 14 days after its application, when the negative effects of drought stress were more evident (Table 7).

**Table 7:** **Effect of Atonik on intensity of photosynthesis, stomatal resistance, chlorophyll content and selected parameters of chlorophyll *a* fluorescence of *A. thaliana* L. plants grown under optimal and drought stress conditions**.

**MWC (%)**	**Term (Days)**	**Photosynthesis (μmol CO_2_ m^−2^ s^−1^)**		**Stomatal resistance (s cm^−1^)**		**Chlorophyll content (relative values)**		**Fv/Fm**		**P.I**.	
		**−Atonik**	**+Atonik**	**−Atonik**	**+Atonik**	**−Atonik**	**+Atonik**	**−Atonik**	**+Atonik**	**−Atonik**	**+Atonik**
60	7	7.34 (± 0.17)	8.97^*^ (± 0.12)	4.85 (± 0.51)	0.86^*^ (± 0.02)	11.25 (± 0.31)	11.61 (± 0.41)	0.832 (± 0.001)	0.833 (± 0.002)	1.77 (± 0.06)	1.67 (± 0.06)
	14	7.57 (± 0.19)	10.38^*^ (± 0.05)	1.83 (± 0.04)	0.44^*^ (± 0.01)	11.31 (± 0.85)	11.08 (± 0.98)	0.828 (± 0.001)	0.828 (± 0.001)	2.79 (± 0.06)	2.69 (± 0.07)
50	7	6.77 (± 0.24)	10.54^*^ (± 0.22)	6.07 (± 0.52)	0.72^*^ (± 0.02)	10.79 (± 0.55)	11.36 (± 0.55)	0.829 (± 0.001)	0.838^*^ (± 0.001)	1.49 (± 0.07)	1.82^*^ (± 0.02)
	14	8.07 (± 0.23)	8.74 (± 0.15)	2.34 (± 0.17)	1.01^*^ (± 0.04)	10.91 (± 1.19)	12.37 (± 1.20)	0.821 (± 0.002)	0.832 (± 0.001)	2.39 (± 0.15)	2.79 (± 0.09)
40	7	9.02 (± 0.23)	9.94 (± 0.34)	2.97 (± 0.35)	0.72^*^ (± 0.01)	12.50 (± 0.32)	12.98 (± 0.35)	0.823 (± 0.003)	0.833 (± 0.001)	1.48 (± 0.07)	1.56 (± 0.05)
	14	7.71 (± 0.37)	8.45 (± 0.43)	3.00 (± 0.31)	1.10^*^ (± 0.13)	10.33 (± 0.75)	10.71 (± 1.26)	0.820 (± 0.002)	0.829 (± 0.002)	2.55 (± 0.17)	2.64 (± 0.14)
30	7	7.19 (± 0.12)	7.21 (± 0.09)	2.52 (± 0.16)	1.02^*^ (± 0.04)	11.45 (± 0.39)	10.92 (± 0.30)	0.827 (± 0.002)	0.815^*^ (± 0.002)	1.56^*^ (± 0.06)	1.08^*^ (± 0.06)
	14	8.87 (± 0.31)	7.40 (± 0.20)	1.47 (± 0.11)	0.97^*^ (± 0.05)	12.54 (± 1.44)	10.03 (± 1.60)	0.818 (± 0.003)	0.825 (± 0.003)	2.66 (± 0.21)	2.26 (± 0.14)
20	7	7.04 (± 0.24)	7.98 (± 0.44)	3.05 (± 0.29)	1.31^*^ (± 0.07)	12.70 (± 0.34)	14.71 (± 0.37)	0.821 (± 0.002)	0.814 (± 0.003)	1.40 (± 0.05)	1.13 (± 0.08)
	14	4.96 (± 0.40)	7.10 (± 0.38)	2.13 (± 0.03)	1.04^*^ (± 0.04)	14.65 (± 2.35)	13.82 (± 1.27)	0.814 (± 0.003)	0.819 (± 0.002)	2.22 (± 0.13)	2.46 (± 0.16)

*Presented data are Mean ± SE, n = 15 (gas exchange and chlorophyll content) or 10 (chlorophyll a fluorescence)*.

* *Values differ significantly at α = 0.05 as determined by LSD of t-Student test*.

The intensity of transpiration increased after Atonik treatment (Table 8). RWC was either only lowered slightly or, at higher drought levels, even increased due to biostimulant application. Plants sprayed with Atonik uptake more water from the medium (Table 8).

**Table 8:** **Effect of Atonik on intensity of transpiration, RWC, and water uptake of *A. thaliana* plants grown under optimal and drought stress conditions**.

**MWC (%)**	**Term (Days)**	**Transpiration (μmol CO_2_ m^−2^ s^−1^)**		**RWC (%)**		**Water uptake (ml pot^−1^)**	
		**−Atonik**	**+Atonik**	**−Atonik**	**+Atonik**	**−Atonik**	**+Atonik**
60	7	2.36 (± 012)	5.55^*^ (± 0.07)				
	14	3.66 (± 0.02)	8.21^*^ (± 0.10)	90.98 (± 0.74)	87.55 (± 1.28)	26.2 (± 0.56)	29.0 (± 0.50)
50	7	1.98 (± 0.10)	6.66^*^ (± 0.20)				
	14	3.63 (± 0.21)	5.40^*^ (± 0.10)	89.11 (± 1.40)	87.77 (± 0.84)	21.5 (± 0.53)	23.0 (± 0.60)
40	7	3.62 (± 0.22)	5.94^*^ (± 0.04)				
	14	3.37 (± 0.19)	5.88^*^ (± 0.32)	92.18 (± 0.66)	90.30 (± 0.31)	21.3 (± 0.45)	22.5 (± 0.41)
30	7	3.50 (± 0.13)	4.99^*^ (± 0.05)				
	14	4.70 (± 0.19)	5.71^*^ (± 0.10)	82.28 (± 0.93)	92.91^*^ (± 0.41)	16.1 (± 0.44)	19.2^*^ (± 0.43)
20	7	3.41 (± 0.17)	4.94^*^ (± 0.15)				
	14	3.78 (± 0.04)	4.78 (± 0.42)	83.22 (± 3.10)	85.55 (± 2.11)	15.2 (± 0.39)	16.3 (± 0.26)

*Presented data are Mean ± SE, n = 15 (intensity of transpiration) or 5*.

* *Values differ significantly at α = 0.05 as determined by LSD of t-Student test*.

#### Optimal and Pt stress conditions

Treatment with Atonik, independently from Pt concentration, had a positive effect on *A. thaliana* plants. The area and number of leaves were greater than in the control by 8.6–15.1 and 0.2–35.5%, respectively (Table 9). Only plants grown in the Pt-free medium had a decreased number of leaves after Atonik application. The biostimulant had a positive effect on biomass accumulation in the aboveground parts of the plants exposed to Pt at concentrations of 2.5 and 25 μM, and the range of this increase amounted to 13–14.5%. In the case of 50 μM, a positive effect was not recorded. Atonik always increased the fresh weight and dry matter of roots (Table 9).

**Table 9:** **Effect of Atonik on the number and area of leaves, and biomass accumulation in *A. thaliana* plants exposed to Pt ions**.

**Combination**	**Number of (leaves plant^−1^)**		**Leaves area (cm^2^ plant^−1^)**		**Fresh weight (g plant^−1^)**				**Dry matter (mg plant^−1^)**			
	**−Atonik**	**+Atonik**	**−Atonik**	**+Atonik**	**Aboveground parts**		**Roots**		**Aboveground parts**		**Roots**	
					**−Atonik**	**+Atonik**	**−Atonik**	**+Atonik**	**−Atonik**	**+Atonik**	**−Atonik**	**+Atonik**
0	143.00 (± 1.61)	128.00 (± 2.92)	222.78 (± 0.74)	271.26 (± 2.17)	8.58 (± 0.50)	9.61 (± 0.17)	2.59 (± 0.12)	2.72 (± 0.02)	880 (± 50)	990 (± 40)	145 (± 12.5)	145 (± 1)
2.5 μM Pt	145.00 (± 3.22)	196.50^*^ (± 3.93)	274.28 (± 7.83)	300.05 (± 3.59)	8.88 (± 0.37)	10.07 (± 0.66)	3.82 (± 0.07)	4.63 (± 0.55)	910 (± 37)	1030 (± 65)	195 (± 2.5)	205 (± 12.5)
25 μM Pt	161.50 (± 8.58)	202.50 (± 1.94)	217.5 (± 3.04)	236.22 (± 3.82)	7.05 (± 0.47)	8.07 (± 0.34)	2.50 (± 0.03)	2.89 (± 0.18)	830 (± 47)	950 (± 34)	140 (± 2)	150 (± 5)
50 μM Pt	193.00 (± 2.29)	193.50 (± 2.51)	219.11 (± 0.34)	252.20 (± 9.44)	7.12 (± 0.64)	6.97 (± 0.50)	2.44 (± 0.22)	2.95 (± 0.34)	850 (± 64)	840 (± 50)	110 (± 5)	140 (± 15)

*Presented data are Mean ± SE, n = 5*.

* *Values differ significantly at α = 0.05 as determined by LSD of t-Student test*.

Intensity of photosynthesis was greater (up to 17.5%) and stomatal resistance was lower (up to 42.5%) in Atonik-treated plants (Table 10). The biostimulant also had a positive effect on the chlorophyll content in leaves, which was higher by 5.1–13.0%. Treatment with Atonik raised the values of Fv/Fm and P.I. in plants exposed to Pt ions (Table 10).

**Table 10:** **Effect of Atonik on intensity of photosynthesis, stomatal resistance, chlorophyll content and selected parameters of chlorophyll *a* fluorescence (Fv/Fm and P.I.) of *A. thaliana* plants exposed to Pt ions**.

**Combination**	**Photosynthesis (μmol CO_2_ m^−2^ s^−1^)**		**Stomatal resistance (s cm^−1^)**		**Chlorophyll content (relative values)**		**Fv/Fm**		**P.I**.	
	**−Atonik**	**+Atonik**	**−Atonik**	**+Atonik**	**−Atonik**	**+Atonik**	**−Atonik**	**+Atonik**	**−Atonik**	**+Atonik**
0	13.06 (± 0.31)	15.03 (± 0.12)	1.80 (± 0.07)	0.99 (± 0.01)	13.18 (± 0.63)	13.57 (± 0.41)	0.823 (± 0.002)	0.824 (± 0.001)	2.30 (± 0.07)	2.32 (± 0.06)
2.5 μM Pt	14.49 (± 0.22)	16.37 (± 0.48)	1.56 (± 0.09)	0.90^*^ (± 0.03)	15.52 (± 0.35)	17.54 (± 0.52)	0.823 (± 0.002)	0.826 (± 0.002)	2.37 (± 0.07)	2.56 (± 0.07)
25 μM Pt	13.66 (± 0.35)	14.66 (± 0.34)	1.56 (± 0.04)	1.34 (± 0.04)	13.07 (± 0.44)	13.47 (± 0.28)	0.806 (± 0.002)	0.817 (± 0.001)	2.09 (± 0.09)	2.17 (± 0.08)
50 μM Pt	12.05 (± 0.34)	14.16^*^ (± 0.33)	2.00 (± 0.16)	1.95 (± 0.11)	12.46 (± 0.55)	13.10 (± 0.65)	0.793 (± 0.004)	0.804 (± 0.002)	1.75 (± 0.11)	1.92 (± 0.10)

*Presented data are Mean ± SE, n = 15 or 10 (chlorophyll a fluorescence)*.

* *Values differ significantly at α = 0.05 as determined by LSD of t-Student test*.

Treatment with Atonik always increased the intensity of transpiration, which was especially evident in 2.5 μM of Pt (Table 11). Effect of Atonik on RWC was marginal as the biostimulant increased this parameter by 3–4% in two lower Pt concentrations, and decreased it by 2% in the highest (Table 11).

**Table 11:** **Effect of Atonik on the intensity of transpiration, RWC, and membrane injuries of *A. thaliana* plants exposed to Pt ions**.

**Combination**	**Transpiration (μmol CO_2_ m^−2^ s^−1^)**		**RWC (%)**		**Membrane injuries (% of control)**			
	**−Atonik**	**+Atonik**	**−Atonik**	**+Atonik**	**Roots**		**Leaves**	
					**−Atonik**	**+Atonik**	**−Atonik**	**+Atonik**
0	4.75 (± 0.22)	5.98 (± 0.10)	85.57 (± 0.77)	82.15 (± 1.28)	0.00	7.58	0.00	2.74
2.5 μM Pt	4.79 (± 0.26)	7.10^*^ (± 0.14)	85.75 (± 1.12)	89.32 (± 0.58)	21.40	7.64	4.41	3.86
25 μM Pt	4.95 (± 0.09)	5.35 (± 0.10)	88.47 (± 0.69)	90.69 (± 0.76)	22.33	12.81	4.95	3.70
50 μM Pt	4.32 (± 0.19)	4.41 (± 0.17)	90.27 (± 1.29)	88.81 (± 0.60)	31.59	19.21	8.50	6.83

*Presented data are Mean ± SE, n = 15 (intensity of transpiration) or 5 (RWC and membrane injuries)*.

* *Values differ significantly at α = 0.05 as determined by LSD of t-Student test*.

Membrane injuries were reduced in biostimulant treated plants (Table 11). After application of Atonik, the level of membrane injuries decreased by 9.5–13.8% in roots and 0.5–1.7% in leaves (Table 11).

## Discussion

### Effect of Atonik on growth and development

The results of this study have clearly demonstrated that Atonik affects all stages of plant development. Changes caused by the biostimulant application are recorded from seed germination and seedling growth (our other study on *Atriplex hortensis, Lolium perenne*, and *Sinapis alba*, data not shown) through the whole ontogenesis. The positive effect of Atonik on germination and seedling growth has been reported by Djanaguiraman et al. (2005a) and Kozak et al. (2008b). This can be explained by the fact that phenolic compounds, which are components of Atonik, interact with gibberellins, which promote seed germination (Taiz and Zeiger, 2002). Fully developed plants treated with a biostimulant are more advanced in growth and development, which has been shown in this work on *A. thaliana* and oilseed rape. *A. thaliana* plants had an increased leaf area and better-developed root system. The stimulation of elongative growth, as a result of the application of Atonik, might be attributed to the greater concentration and/or activity of auxins (Djanaguiraman et al., 2004a, 2005b). Plants treated with a biostimulant are characterized as having a higher inhibition of IAA oxidase, which ensures greater activity of naturally synthesized auxins (Stutte and Clark, 1990) and a greater number of high-affinity binding sites of IAA (Libbenga and Mennes, 1987). Feverfew (Gruszczyk and Berbeć, 2004), cotton (Djanaguiraman et al., 2005a, 2009), tomato (Djanaguiraman et al., 2004b, 2005a), maize (Michalski et al., 2008), and soya (Kozak et al., 2008a) are all taller after the Atonik application. The biostimulant stimulates the growth of shoots in sweet pepper (Panajotov et al., 1997) and roots in cotton (Djanaguiraman et al., 2005a) and ginseng (Kołodziej, 2004). The promotion of leaf development is noted in cotton and tomato (Djanaguiraman et al., 2005b, 2009) and sweet pepper (Panajotov et al., 1997). Other biostimulants also stimulate plant growth. For example bio-algeen S90 increases the height of tomato plants (Dobromilska and Gubarewicz, 2008). The length of shoots has been positively influenced by various biostimulants in bell pepper, raspberry, and apple (Basak and Mikos-Bielak, 2008; Ochmian et al., 2008; Stępowska, 2008a). Bio Jodis, Goëmar Goteo, Bio-algeen S90 and Resistim stimulate root growth in tomato (Kossak and Dyki, 2008). A greater number of leaves and/or their area have been recorded in tomato treated with Bio-algeen S90 (Dobromilska and Gubarewicz, 2008), apple with Kelpak (Basak and Mikos-Bielak, 2008) and bell pepper with four different biostimulants (Stępowska, 2008a).

However, in literature there is also data indicating a lack of positive effects of biostimulants on plant growth. Malarz et al. (2008) demonstrate the marginal influence of Atonik on the height of spring rape. Atonik did not affect the growth of oilseed rape (Budzyński et al., 2008), bell pepper (Csizinszky, 2001), or maize (Księżak, 2008) at all.

Atonik-treated plants are more advanced in generative development. In this study, the biostimulant increased the number of inflorescences, pods and seeds. This was true for *A. thaliana* and oilseed rape, irrespective of whether the plants were grown in the field or in growth chambers, no matter if under optimal or stress conditions. These results confirmed previous findings by Budzyński et al. (2008) and Malarz et al. (2008), who also demonstrate the positive effect of Atonik on the generative development of oilseed rape. Atonik also increases the number of pods and seeds in soya (Gulluoglu et al., 2006; Kozak et al., 2008a), flowers and bolls in cotton (Djanaguiraman et al., 2005b), flowers and fruits in tomato (Djanaguiraman et al., 2004a), and inflorescences in feverfew (Gruszczyk and Berbeć, 2004). Above corresponds well with works of Górnik and Grzesik (2002, 2005), who found that Atonik improves the generative development of China aster, but only when applied during flowering. A greater number of flowers and fruits has also been reported in tomato and apple plants treated with Bio-algeen S-90 and Frigocur, respectively (Basak and Mikos-Bielak, 2008; Dobromilska and Gubarewicz, 2008). Goëmar BM 86 stimulates fruit growth in pears (Błaszczyk, 2008) and ripening in raspberries (Krok and Wieniarska, 2008).

In contrast, Krawiec (2008) found an ambiguous effect of simultaneous treatment with Goëmar BM 86 and Atonik on the number of fruits in chokeberries. Atonik did not affect the size and diameter of strawberry fruits (Miranda-Stalder et al., 1990) or the number of grains in the cob and size of the cob in maize (Księżak, 2008).

### Effect of Atonik on biomass accumulation and yielding

This study's results have shown, that the faster growth and development of Atonik-treated plants is associated with a greater biomass accumulation. After the application of the biostimulant, the fresh weight and dry matter of whole *A. thaliana* and oilseed rape plants, as well as their particular organs, were greater. In the case of oilseed rape, this effect was more pronounced in the 2007 vegetative season in which plants experienced a spring frost. It is worth mentioning that the increase of biomass accumulated in generative organs was greater than in vegetative ones, which also supported the hypothesis mentioned above concerning the promotion of generative development. A greater biomass accumulation in oilseed rape sprayed with Atonik has also been recorded by Bečka et al. (2004). Similar results are recorded for cotton and tomato (Djanaguiraman et al., 2004b, 2005a), goldenrod (Kołodziej, 2008), *Amaranth* sp. (Wrochna et al., 2008), and common osier (Harasimowicz-Hermann and Czyż, 2008). A stimulation of dry-matter accumulation in the roots and aboveground organs of oilseed rape treated with Route has been reported by Krawczyk and Skoczyński (2008). Bio-algeen S-90 increases the dry matter of tomato fruits (Dobromilska and Gubarewicz, 2008) and Goëmar Goteo positively affects biomass accumulation in lettuce (Kowalczyk and Zielony, 2008) and nappa cabbage (Gajewski et al., 2008). Stępowska (2008a) has recorded a greater weight of whole plants and separately of roots and leaves in bell pepper treated with different biostimulants. The increase in biomass accumulation resulting from biostimulant treatment is not usually very spectacular and ranges from just a little to 20%, but much higher values are also reported, as in the case of feverfew and ginseng plants in which the application of Atonik results in the increase in biomass of 54 and 51.5% (fresh weight and dry matter) and 43 and 61% (fresh weight of roots and aboveground organs) respectively (Gruszczyk and Berbeć, 2004; Kołodziej, 2004).

The increased biomass accumulation after Atonik application usually resulted in a higher yield. In this study the biostimulant increased the yield of oilseed rape, but only when it was applied as a single spray. This has also been shown in oilseed rape by Budzyński et al. (2008) and Malarz et al. (2008), as well as in many other species, such as beetroot (Černý et al., 2002; Kositorna and Smoliński, 2008), potato (Czeczko and Mikos-Bielak, 2004; Sawicka and Mikos-Bielak, 2008), cotton (Djanaguiraman et al., 2005b, 2009; Bynum et al., 2007), maize (Michalski et al., 2008), soya (Kozak et al., 2008a), tomato (Djanaguiraman et al., 2004a,b; Gajc-Wolska et al., 2010), apple (Basak and Mikos-Bielak, 2008), common chicory (Černý et al., 2008), leek and celery (Czeczko and Mikos-Bielak, 2004), and raspberries (Grajkowski and Ochmian, 2007). Other biostimulants also increase yield and this has been reported for a great number of crops, such as apple, bell pepper, cereals, lettuce, lupine, maize, mustard, nappa cabbage, pea, potato, raspberry, and strawberry (Abetz and Young, 1983; Dobromilska and Gubarewicz, 2008; Gajewski et al., 2008; Kossak and Dyki, 2008; Kowalczyk and Zielony, 2008; Matysiak and Kaczmarek, 2008; Ochmian et al., 2008; Sas-Paszt et al., 2008; Stępowska, 2008b; Wrona and Misiura, 2008; Khan et al., 2009).

However, there are reported studies showing that biostimulants have either a minor, no influence on yield or even a negative effect. The lack of a positive effect of Atonik on yield has been recorded here in cucumber and earlier reported by Miranda-Stalder et al. (1990), Csizinszky (2001), Krawiec (2008), and Księżak (2008) in strawberries, bell pepper, chokeberries and maize. Basak and Mikos-Bielak (2008) showed that Frigocur, Kelpak, and Help even negatively affect the yield of apples.

The effect of biostimulants on biomass accumulation and yield may depend on a number of environmental factors. In literature the emphasis is on the influence of the cultivar, preparation concentration and term of its application, growing conditions, fertilization employed, and location (Basak and Mikos-Bielak, 2008; Łyszkowska et al., 2008; Maciejewski et al., 2008; Sas-Paszt et al., 2008; Gajc-Wolska et al., 2009; 2012).

### Effect of Atonik on photosynthetic apparatus

Stimulated biomass production and yield recorded for many species are attributed to a more efficient photosynthetic apparatus in plants sprayed with Atonik. This has been shown in this study for *A. thaliana* and oilseed rape. Plants treated with Atonik had higher (i) leaf area, (ii), chlorophyll content, (iii) intensity of photosynthesis, and (iv) values of chlorophyll *a* fluorescence parameters. In our preliminary studies on wheat also increase in LAI (Leaf Area Index, data not shown) was recorded.

In this work, the biostimulant increased the chlorophyll content in both *Brassicaceae* species examined and under all experimental conditions. It is worth mentioning that this increase in the case of oilseed rape was more evident at the end of the growing season, which may suggest that Atonik either promotes *de novo* chlorophyll biosynthesis or slows down its degradation, delaying the aging processes. A similar result was reported by Djanaguiraman et al. (2009) in cotton. A greater chlorophyll content in plants treated with Atonik was recorded also in common osier (Wróbel and Woźniak, 2008), *Amaranthus* sp. (Wrochna et al., 2008) and cotton (Djanaguiraman et al., 2009). Four different biostimulants increased the content of chlorophyll in bell pepper (Stępowska, 2008a). In contrast to the above, Kowalczyk et al. (2008) did not find that Atonik and Aminoplant had a positive effect on the content of chlorophyll in lettuce, and Krajewska and Latkowska (2008) even demonstrated a reduction of chlorophyll content in hosta and bergenia treated with Siapton.

In *A. thaliana* and oilseed rape plants treated with Atonik, the intensity of photosynthesis was greater, which is in the line with the results of Borowski and Blamowski (2009), Wróbel and Woźniak (2008) and Djanaguiraman et al. (2009) on basil, common osier and cotton. A new discovery from this study has been that the positive effect of Atonik on the intensity of photosynthesis may last up to 7 weeks, which is much longer than previously believed. According to the manufacturer of Atonik, its working time was estimated to be a maximum of 2–3 weeks. A higher intensity of photosynthesis could be explained, at least partially, by lowered stomatal resistance (or increased stomatal conductance), which ensures an easier and greater CO_2_ flow to chloroplast. Increased stomatal conductance has been reported for basil plants treated with Atonik (Borowski and Blamowski, 2009) and cotton with PGR-IV (Zhao and Oosterhuis, 1997). Atonik accelerates the transport of photoassimilates within cells and between them to various tissues and organs (Yamaki et al., 1953). Wilson and Kaczmarek (1993) show that the phosphorylated form of sodium para-nitrophenolate reduces the activity of cation channels (Ca^2+^, K^+^, and Na^+^) by inhibiting the activity of the enzyme tyrosine phosphatase. A decreased activity in the cation channel causes the reduction of Ca^2+^ concentration in the cells, which results in the increase of cytoplasm movement (Roberts and Harmon, 1992). The above is in line with Oosterhuis and Robertson (2000), who suggest that the increased photosynthesis in cotton treated with PGR-IV is related to a quicker transport of assimilates from its source (leaves) to various sinks.

Atonik also has a positive effect on the parameters of chlorophyll *a* fluorescence. The values of Fv/Fm and P.I were usually higher in Atonik-treated *A. thaliana* plants. The positive effect of this biostimulant on chlorophyll *a* fluorescence has previously been reported by Djanaguiraman et al. (2009) in cotton. In contrast to the above, biowska (Gawlik and Gołębiowska (2008) record decreased values of Fv/Fm in pea plants sprayed with humic acids.

It should be pointed out that although the level of beneficial influence on particular/parameters of the photosynthetic apparatus is not very spectacular, it has to be taken into consideration that they “work additively.” Photosynthesis takes place over several hours a day during most of the sunny days of the vegetation season, which, together with the positive effects on other processes, substantially contributes to greater final plant productivity.

### Effect of Atonik on plant water status

Application of Atonik also affects a plant's water status. The lowered stomatal resistance earlier discussed leads to higher intensity of transpiration in *A. thaliana* and oilseed rape plants, as reported by Wróbel and Woźniak (2008), Borowski and Blamowski (2009), and Zhao and Oosterhuis (1997). Increased transpiration intensity means greater water loss by plants and, as a consequence, it can be expected that RWC should be lower, especially in *A. thaliana* plants grown under drought stress conditions. Contrary to this expectation, RWC was almost unchanged or, in some cases, even slightly higher. This result can be explained by the improved water uptake after Atonik application, as shown here by daily pot weighing, which is related to a better-developed root system, both in terms of length and biomass. Improved RWC in biostimulant-sprayed plants has also been reported by Wrochna et al., (2008) in *Amaranthus* sp. and Wróbel and Woźniak (2008) in common osier.

It is worth noticing that simultaneously with more efficient water uptake from soil, plants are also taking up more nutrients, as demonstrated by Stutte et al. (1987) and Oosterhuis (2008).

### Effect of Atonik on plant quality

Atonik changes the chemical composition of cucumber fruits, positively in the case of soluble solids and phosphorus, but negatively in terms of nitrates and calcium. In literature there is data that the application of Atonik increases the content of carbohydrates (Czeczko and Mikos-Bielak, 2004; Djanaguiraman et al., 2005a; Kositorna and Smoliński, 2008), crude fat (Malarz et al., 2008), amino acids (Djanaguiraman et al., 2005a) proteins (Czeczko and Mikos-Bielak, 2004; Djanaguiraman et al., 2005a; Oosterhuis, 2008), but decreases the level of nitrates (Kowalczyk et al., 2008). On the other hand, Atonik may decrease the concentration of vitamin C (Czeczko and Mikos-Bielak, 2004; Grajkowski and Ochmian, 2007).

### Effect of Atonik on the mitigation of stress effects

There is common opinion that Atonik mitigates effect of stress conditions. This study proved that the application of Atonik diminished the negative impact of drought and noble metal stresses in *A. thaliana* and enhanced the recovery from the late spring frost in oilseed rape. *A. thaliana* plants grown with a water deficit and Pt stresses and treated with a biostimulant had accelerated growth and development, accumulated more biomass and all studied physiological processes were stimulated. Protective effect of Atonik was especially evident in the case of photosynthetic apparatus. For example in oilseed rape grown in the 2007 season, when the late spring frost occurred, Atonik improved chlorophyll *a* fluorescence parameters. Chlorophyll *a* fluorescence is informative tool to analyze and understand plant's response to fluctuations in environmental conditions. Higher intensity of photosynthesis was recorded in *A. thaliana* plants grown under drought conditions. One of the first responses of plants to drought stress is the closing of stomata, a process controlled by, among other, ABA (Blatt, 2000; Schroeder et al., 2001; Shinozaki and Yamaguchi-Schinozaki, 2007). A decreased level of free ABA after application of Atonik has been shown in other studies conducted by the authors in *A. thaliana* plants grown with water deficit (Przybysz et al., 2010). Changes in ABA regulation by lowering its concentration resulted in more efficient gas exchange and stimulated growth in stress conditions recorded in this work.

More evidence that Atonik protects plants against the negative effects of stress was shown in this work in the decreased level of plasma membrane injuries caused by Pt, both in the roots and leaves of *A. thaliana*. Similar results were obtained in previous work on plants exposed to Cd^2+^ (Gawrońska et al., 2008). The reduction of membrane injuries in the case of both metals was more pronounced in roots, which were in direct contact with toxic elements. A decrease in plasma membrane injuries has also been found in Atonik-treated *Amaranthus* sp. (Wrochna et al., 2008), basil (Borowski and Blamowski, 2009), and cotton (Djanaguiraman et al., 2009).

The protective role of Atonik has also been recorded in the case of heavy metals in the example of Cd^2+^ (Gawrońska et al., 2008), salinity (Wrochna et al., 2008), spring frost (Basak and Mikos-Bielak, 2008), and heat (Gulluoglu et al., 2006). Górnik et al. (2007) and Górnik and Grzesik (2008) recorded an increased tolerance of grape cuttings to extreme temperatures and water deficit after treatment with a few biostimulants. Since many defense mechanisms against different unfavorable conditions, especially of abiotic origin, are very much the same, it can be assumed that Atonik probably also decreases the negative effects of other stresses not mentioned in this work.

Most of the stresses may induce the appearance of excessive amounts of reactive oxygen species (ROS) and consequently the exacerbation of oxidative stress (Iturbe-Ormaetxe et al., 1998). Discussed above reduction of membrane injuries may reduce creating of ROS in plants. Moreover, it has been reported that the application of Atonik contributes to a decreased level of oxidative stress by increasing (i) the activity of anti-oxidizing system enzymes: ascorbate peroxidase, catalase, glutathione reductase, and (ii) total antioxidative capacity to a greater extent than the increase in anion-radical level (Wrochna et al., 2008; Djanaguiraman et al., 2004a, 2005a,b, 2009). Atonik also positively affects the production of proline and polyols, two important compatible metabolites involved in anti-stress mechanisms (Djanaguiraman et al., 2004b, 2009).

All changes presented above have probably their origin in modified, after Atonik treatment, profile of gene expression. In the literature some studies report that after the application of biostimulants expression of genes related to defense mechanism is upregulated. The treatment of *A. thaliana* plants exposed to freezing stresses with algae extract result in changes of expression in about 5% (1113) of all *A. thaliana* genes (Nair et al., 2012). About 2% (463 genes) of the differentially expressed genes are upregulated and 3% (650 genes) downregulated. The authors report that some of these genes were involved in the plant's defense mechanisms (Nair et al., 2012). The application of algal extracts prior to pathogen infection in alfalfa cause upregulation of 152 genes, mostly plant defense genes, such as those involved in phytoalexin, PR proteins, cell wall proteins, and oxylipin pathways (Cluzet et al., 2004). In *A. thaliana* grown under salt stress and treated with Aminoplant, Cambri et al. (2008) demonstrate changes in expression of a few genes responsible for the plant's defense mechanisms.

There is a commonly held view, as also demonstrated in this work, that the positive impact of biostimulants is more evident and that the potential of these compounds can be fully exploited only when plants are grown under stressful conditions, while under optimal conditions their positive effect is sometimes marginal (Budzyński et al., 2008; Krawiec, 2008; Maciejewski et al., 2008) or even not reported at all (Csizinszky, 2001). Possible protective effect of biostimulants depends also on many other, not discussed here factors, mostly the level and duration of stresses and moment of Atonik application.

## Conclusions

The biostimulant Atonik affects every level of a plant's biological organization in terms of structure and function, from canopy and whole plant, *via* particular organs and cells, to physiological and biochemical processes.

1. Atonik stimulates plant growth and development, particularly generative.
2. Biomass accumulation, both fresh weight and dry matter, and yield production are stimulated by Atonik due to a higher efficiency of the photosynthetic apparatus manifested by (i) a higher leaf area, (ii) a higher chlorophyll content, (iii) greater intensity of photosynthesis, and (iv) an improvement of chlorophyll *a* fluorescence parameters.
3. Despite higher transpiration and lower stomatal resistance, RWC was unchanged in Atonik-treated plants due to the promotion of root development and consequently an increased water uptake.
4. The effect of Atonik on the quality and chemical composition of fruits was diverse and depended on the parameter measured and cultivar examined.
5. The application of Atonik played simulative role under optimal conditions and protective against spring frost, drought, and noble metal stresses.
6. The positive effect of Atonik is much more pronounced when plants are growing under stress conditions.

### Conflict of interest statement

The authors declare that the research was conducted in the absence of any commercial or financial relationships that could be construed as a potential conflict of interest.
